# Detection of quantitative trait loci for mineral content of Nelore *longissimus dorsi* muscle

**DOI:** 10.1186/s12711-014-0083-3

**Published:** 2015-03-11

**Authors:** Polyana C Tizioto, Jeremy F Taylor, Jared E Decker, Caio F Gromboni, Mauricio A Mudadu, Robert D Schnabel, Luiz L Coutinho, Gerson B Mourão, Priscila SN Oliveira, Marcela M Souza, James M Reecy, Renata T Nassu, Flavia A Bressani, Patricia Tholon, Tad S Sonstegard, Mauricio M Alencar, Rymer R Tullio, Ana RA Nogueira, Luciana CA Regitano

**Affiliations:** Department of Genetics and Evolution, Federal University of Sao Carlos, São Carlos, SP Brazil; Division of Animal Sciences, University of Missouri, Columbia, MO USA; Federal Institute of Education, Bahia Science and Technology, Valença, BA Brazil; Embrapa Southeast Livestock, São Carlos, SP, Brazil; Department of Animal Science, University of São Paulo/ESALQ, Piracicaba, SP Brazil; United States Department of Agriculture (USDA), Agricultural Research Service, Beltsville, MD USA; Department of Animal Science, Iowa State University, Ames, IA USA

## Abstract

**Background:**

Beef cattle require dietary minerals for optimal health, production and reproduction. Concentrations of minerals in tissues are at least partly genetically determined. Mapping genomic regions that affect the mineral content of bovine *longissimus dorsi* muscle can contribute to the identification of genes that control mineral balance, transportation, absorption and excretion and that could be associated to metabolic disorders.

**Methods:**

We applied a genome-wide association strategy and genotyped 373 Nelore steers from 34 half-sib families with the Illumina BovineHD BeadChip. Genome-wide association analysis was performed for mineral content of *longissimus dorsi* muscle using a Bayesian approach implemented in the GenSel software.

**Results:**

Muscle mineral content in *Bos indicus* cattle was moderately heritable, with estimates ranging from 0.29 to 0.36. Our results suggest that variation in mineral content is influenced by numerous small-effect QTL (quantitative trait loci) but a large-effect QTL that explained 6.5% of the additive genetic variance in iron content was detected at 72 Mb on bovine chromosome 12. Most of the candidate genes present in the QTL regions for mineral content were involved in signal transduction, signaling pathways via integral (also called intrinsic) membrane proteins, transcription regulation or metal ion binding.

**Conclusions:**

This study identified QTL and candidate genes that affect the mineral content of skeletal muscle. Our findings provide the first step towards understanding the molecular basis of mineral balance in bovine muscle and can also serve as a basis for the study of mineral balance in other organisms.

**Electronic supplementary material:**

The online version of this article (doi:10.1186/s12711-014-0083-3) contains supplementary material, which is available to authorized users.

## Background

Genome-wide association studies (GWAS) can be used to identify quantitative trait loci (QTL) associated with complex traits and to better understand which genes and biological mechanisms underlie phenotypic variation. Knowledge on the genes that affect mineral content in beef cattle would provide valuable insights into ruminant metabolic diseases [1], assist in animal diet formulation, and allow the development of beef products that improve human nutrition. Beef cattle require many dietary minerals for optimal health, production and reproduction. There are two categories of minerals, i.e., macrominerals that are needed in larger amounts and trace minerals. Diets that do not provide adequate amounts of these minerals can lead to animal health problems and losses in profitability for producers. However, because trace minerals are required only in very small amounts, inadequate supplementation may have negative effects on animal health.

Previous studies on the Nelore cattle population used here [2,3] and on other *Bos taurus* populations [4] have suggested that muscle mineral content may influence beef quality traits, such as meat tenderness, possibly because the effect of the genes that control these traits depends on the presence of specific minerals. For example, reduced calcium availability may affect the calpain-calpastatin system, which plays a major role in *postmortem* proteolysis and meat tenderization [5-7], as well as being crucial for muscle contraction.

The concentrations of minerals in the muscle depend on mineral supplementation and excretion [1], but can also be affected by environmental effects such as birthplace, age and breed [3]. Furthermore, it has been shown that mineral content in tissues is in part genetically determined and heritable [8,9] and that the genes involved may act via receptor, transporter and chaperone proteins [1].

Thus, a genome-wide association study of the mineral content in beef muscle was undertaken to identify regions in the bovine genome that control variation in mineral balance, transportation, absorption and excretion. We identified QTL regions that contain genes that could be related to variation in the amounts of arsenic (As), calcium (Ca), chromium (Cr), cobalt (Co), copper (Cu), iron (Fe), magnesium (Mg), manganese (Mn), phosphorus (P), potassium (K), selenium (Se), sodium (Na), sulfur (S) and zinc (Zn), in the *longissimus dorsi* muscle of Nelore cattle.

## Methods

### Animals

The research protocol was approved by the Embrapa Southeast Livestock (São Carlos, São Paulo, Brazil) ethics committee. Half-sib families, totaling 373 Nelore steers, were produced from 34 sires chosen to represent the main commercial lineages in Brazil, i.e., different genealogies, and based on the average price of semen used by Brazilian beef cattle producers in order to sample bulls widely used in the country. These half-sib families were produced during two different breeding seasons by artificial insemination of commercial and purebred Nelore dams with semen from the 34 purebred Nelore sires. On average, 20 offspring per sire were produced of which the steer progeny were used in this study.

Calves were born and raised on four farms and transported to a feedlot at Embrapa Southeast Livestock when they were about 21 months of age, where they were maintained in individual (n = 158) or collective pens (n = 215, with 10 animals per collective pen) and allowed *ad libitum* access to feed and water. The adaptation period to the diet, before allocation to the pens, was approximately 28 days. Animals were fed twice daily. Diets contained 40% dry matter (DM) in the form of corn silage with 13.5% crude protein; and energy densities of 2.8 Mcal metabolizable energy per kg DM. The remaining 60% of DM was in the form of concentrate, and contained ground corn, soybean meal, cotton seed, soybean hulls, limestone, a mineral mixture, urea and monensin (Rumensin®). Steers were slaughtered after about 90 days of feeding at a small-scale slaughter and processing facility at an average age of 734 days and a mean weight of 383.2 kg when backfat thickness reached about 5 mm, which was evaluated by ultrasound.

### DNA extraction and genotyping

Five mL blood samples were collected from each steer and DNA extractions were performed using a salting out method [10]. All animals were genotyped using the Illumina BovineHD BeadChip (Illumina Inc., San Diego, CA) either at the USDA ARS Bovine Functional Genomics Laboratory in Beltsville (MD) or at the ESALQ Genomics Center, Piracicaba, São Paulo, Brazil. Genotypes were called using the Illumina Genome Studio software [11]. Samples were filtered based on a call rate [12] greater than 85% and heterozygosity greater than 40%. Loci were deleted if their Illumina probe sequence could not be uniquely mapped to an autosome on the UMD3.1 reference assembly [13], if their call rate was less than 90%, minor allele frequency was less than 0.5%, or if they deviated severely from Hardy Weinberg equilibrium (χ^2^ > 100.0), as described by Tizioto et al. [2]. Only autosomal genotypes were used in the association analysis.

### Phenotype collection

At slaughter, 2.5 cm thick steaks were sampled as a cross section of the *longissimus dorsi* muscle between the 11^th^ and 13^th^ ribs. Mineral phenotypes were measured as described by Tizioto et al. [3]. The *longissimus dorsi* muscle was used because it is a major commercialized beef cut and is conventionally used for measuring most meat quality traits [14]. Briefly, an entire steak was lyophilized; homogenized and 100 mg was sampled for chemical analyses. Analytical grade reagents and ultrapure water (Milli-Q system, Millipore, Billerica, MA, USA) were used and standard solutions were certified with plasma grade and high purity materials from SpecSol (Jacareí, SP, Brazil). A closed-vessel microwave digestion system (Ethos-1600, Milestone-MLS, Sorisole, Italy) equipped with optic fiber temperature and pressure sensors was used for digestion of the sample. *Longissimus dorsi* muscle samples were digested in the microwave vessel using 2 mL of sub-boiled concentrated HNO_3_, 2 mL of H_2_O_2_ (30% w/w) and 6.0 mL of ultrapure water in closed vessels made of perfluoroalkoxy copolymer resin (PFA). A three-step heating cycle was applied: (1) a first heating ramp at 120°C (1300 W) for 10 min; (2) a second heating ramp at 170°C (1500 W) for 15 min; and (3) a heat treatment at 170°C for 35 min.

After digestion, samples and blank solutions were transferred to 10.0 mL volumetric flasks and volumes were completed with ultrapure water. The mineral contents were determined by Vista Pro-CCD ICP-OES spectrometer with radial view (Varian, Mulgrave, Australia). Excitation wavelengths were chosen to minimize spectral interference and produce the highest intensity emission for each element. A linear calibration was calculated with up to five points from preparations using standard analytical solutions.

Accuracy and precision of the method used to measure the mineral amounts were evaluated by measuring the recovery level and relative standard deviation of the certified reference materials Bovine Liver 1557b and Bovine Muscle 8414 (N = 3) from the National Institute of Standards and Technology (Gaithersburg, MD, USA). The experiment was carried out in triplicate. Recovery values were calculated according to the values obtained for the certified reference samples.

### Genome-wide association analysis

Missing genotypes were imputed using BEAGLE v3.3.2 [15] without the use of pedigree information. *Longissimus dorsi* muscle mineral content was analyzed under a Bayesian model using GenSel software [16], as explained below, using an approach similar to that described by Tizioto et al. [2] to identify genomic regions influencing these phenotypes. First, BayesC [17], was used to estimate additive genetic and residual variances, assuming a π parameter (prior distribution for θ) value of 0 (i.e., assuming that all SNPs contributed to explaining genetic variance in each trait). From these estimates the phenotypic variance was estimated as the sum of the additive genetic and residual variance components and the heritability as the ratio of additive genetic to phenotypic variances. The estimates of additive genetic and residual variance from the BayesC analyses were then used to run the BayesB analyses [18] to estimate SNP effects. The parameter π was set to 0.9995 such that the number of SNP effects to be estimated was less than the number of animals with data (449 364 × (1 - π) = 224 SNPs), as suggested by Mateescu et al. [9]. The BayesB analysis fits separate variances for each SNP in the model, which allows large-effect SNPs to be estimated without excessively regressing their effects towards 0. The statistical model included the fixed effects of six contemporary groups that were formed using combinations of birth (n = 4) and feedlot location (n = 2) and breeding season (n = 2). The animal’s age at slaughter was included as a covariate.

The genomic regions that included the 224 SNPs that had the largest posterior probability [19] of being incorporated in the model for each trait were examined separately for candidate genes within ± 100 kb of each SNP using Map Viewer (http://www.ncbi.nlm.nih.gov/mapview/). The enriched pathways and functional gene clusters that involved the candidate genes within the associated genomic regions were identified using the Database for Annotation, Visualization and Integrated Discovery (DAVID) software [20]. In addition to estimating single SNP effects, Gensel software estimates the proportion of additive genetic variance that is explained by each 1-Mb genomic window for each trait which was considered to be a QTL in this study and it was also used to identify potential candidate genes.

## Results

### Summary statistics for mineral content

Certified reference materials were evaluated to ensure the accuracy of the sample preparation procedure. The recovery values and triplicate deviations of the minerals in certified reference muscle samples ranged from 92.3 to 127.3% and 2 to 11.2, respectively. Reasonable agreement was observed between samples and the certified values [21], which indicated the effective recovery of analytes after digestion and their accurate detection.

The raw means, variance components and heritability estimates generated by the BayesC analyses are in Table 1. Heritability estimates for all mineral contents were medium to high (0.29 – 0.36).

**Table 1:** **Raw means, standard deviations, and estimates of heritability and variance components for the content of each evaluated mineral in the** ***longissimus dorsi*** **muscle of 373 Nelore steers**

**Trait**	**Mean (mg/kg) ± SE**	**σ** ^2^_e_	**σ** ^2^_a_	**h** ^2^
As	0.4 ± 0.001	0.18	0.09	0.34
Ca	164.3 ± 5.2	0.05	0.02	0.31
Cr	0.4 ± 0.03	0.24	0.13	0.36
Co	0.01 ± 0.0007	0.12	0.06	0.33
Cu	1.8 ± 0.05	0.04	0.02	0.30
Fe	44.7 ± 1.6	0.31	0.15	0.32
Mg	759.5 ± 7.6	0.03	0.01	0.29
Mn	0.19 ± 0.01	0.31	0.16	0.33
P	7356.4 ± 85.4	0.04	0.01	0.29
K	1077.5 ± 13.7	0.04	0.02	0.30
Se	0.2 ± 0.005	0.05	0.02	0.32
Na	1746.3 ± 22.86	0.04	0.02	0.29
S	6287.7 ± 84.6	0.04	0.02	0.30
Zn	79.8 ± 1.2	0.04	0.02	0.31

As = arsenic, Ca = calcium, Cr = chromium, Co = cobalt, Cu = copper, Fe = iron, Mg = magnesium, Mn = manganese, P = phosphorus, K = potassium, Se = selenium, Na = sodium, S = sulfur and Zn = zinc; SE: standard error.

### Genome-wide association study and underlying gene annotations

**Figure 1:**
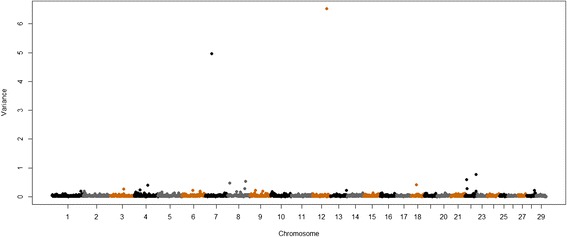
**Genome-wide plot of additive genetic variance explained by 1-Mb marker windows for iron concentration of Nelore** ***longissimus dorsi*** **muscle.**

After filtering SNPs based on call rate, allele frequency and Hardy-Weinberg equilibrium, and imputing 0.80% of missing genotypes, 449 364 loci were available for the GWAS. Based on the number of available markers and the π parameter (0.9995), we calculated the number of SNPs that had the largest posterior probability which were used to perform functional annotation (449 364 × (1 - π) = 224 SNPs).The GWAS identified regions across the genome that harbored QTL for all minerals analyzed in this study. Most of the identified QTL were of small-effect (i.e., explained less than 1% of the additive genetic variance in each trait). A few large-effect QTL were found. A QTL that explained 6.5% of the additive genetic variance for Fe content (Table 2) was detected at 72 Mb on BTA12 (BTA for *Bos taurus* chromosome) (Figure 1). The genomic region that harbored this QTL includes genes from the ATP-binding cassette family (Table 2).

**Table 2:** **Chromosome, location, number of SNPs, and percentage of additive genetic variation explained by the most important QTL regions associated with mineral concentration in muscle tissue of Nelore cattle**

**Trait**	**Chr** ^a^	**Position (Mb)** ^b^	**Nb SNPs** ^c^	**Variance (%)** ^d^	**Candidate genes**
Ar	25	17	227	1.1	*COQ7, TMC7,TMC5, GDE1, CCP110, KNOP1, IQCK, GPRC5B* and *GPR139*
Ca	8	56	126	2.6	*-*
Cr	20	65	267	1.1	*MTRR, FASTKD3, ADCY2* and *TRNAC-ACA*
Cr	9	50	160	1.0	*ASCC3, TRNAC-GCA, SIM1, MCHR2, PRDM13, CCNC* and *USP45*
Co	6	70	300	0.5	*USP46, RASL11B, SCFD2, FIP1L1and LNX1*
Cu	18	29	229	1.1	*TRNAA-AGC* and *CDH8*
Fe	12	72	94	6.5	*TRNAC-GCA*
Fe	7	32	257	5.0	*PRDM6, PPIC, SNX24* and *SNX2*
Mg	10	50	144	1.3	*FOXB1, BNIP2, GTF2A2, GCNT3* and *FAM81A*
Mn	6	66	219	0.7	*GABRG1, GABRA2, TRNAW-CCA* and *COX7B2*
P	3	115	236	1.1	*SH3BP4*
K	10	50	144	1.2	*FOXB1, BNIP2, GTF2A2, GCNT3* and *FAM81A*
Se	11	22	243	3.5	*TMEM178* and *THUMPD2*
Se	12	54	186	1.8	*POU4F1, RNF219, RBM26* and *NDFIP2*
Na	6	35	184	0.4	*FARSB* and *CCSER1*
S	1	28	193	0.7	*TRNAS-GGA and GBE1*
Zn	8	10	218	2.5	*FZD3, FBXO16, ZNF395, PNOC, TRNAG-GCC, ELP3, NUGGC, SCARA5, PBK ESCO2, CCDC25* and *SCARA3*

As = arsenic, Ca = calcium, Cr = chromium, Co = cobalt, Cu = copper, Fe = iron, Mg = magnesium, Mn = manganese, P = phosphorus, K = potassium, Se = selenium, Na = sodium, S = sulfur and Zn = zinc; ^a^Chr = chromosome; ^b^position of the QTL on the chromosome in Mb; ^c^number of SNPs within the 1 Mb window detected as harboring the QTL; ^d^percentage of the additive genetic variance explained by the 1 Mb window estimated by calculating the variance of the molecular breeding values for all animals using the SNP effects for each window.

**Figure 2:**
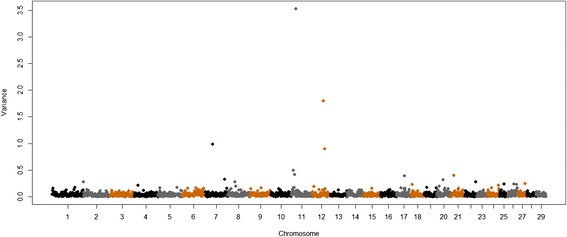
**Genome-wide plot of additive genetic variance explained by 1-Mb marker windows for selenium concentration of Nelore** ***longissimus dorsi*** **muscle.**

**Figure 3:**
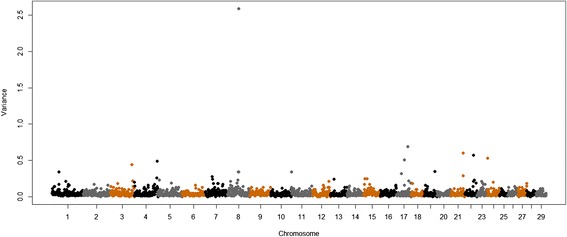
**Genome-wide plot of additive genetic variance explained by 1-Mb marker windows for calcium concentration of Nelore** ***longissimus dorsi*** **muscle.**

**Figure 4:**
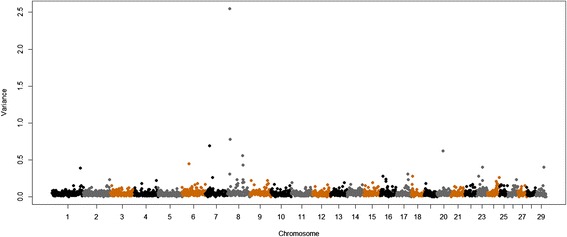
**Genome-wide plot of additive genetic variance explained by 1-Mb marker windows for zinc concentration of Nelore** ***longissimus dorsi*** **muscle.**

**Figure 5:**
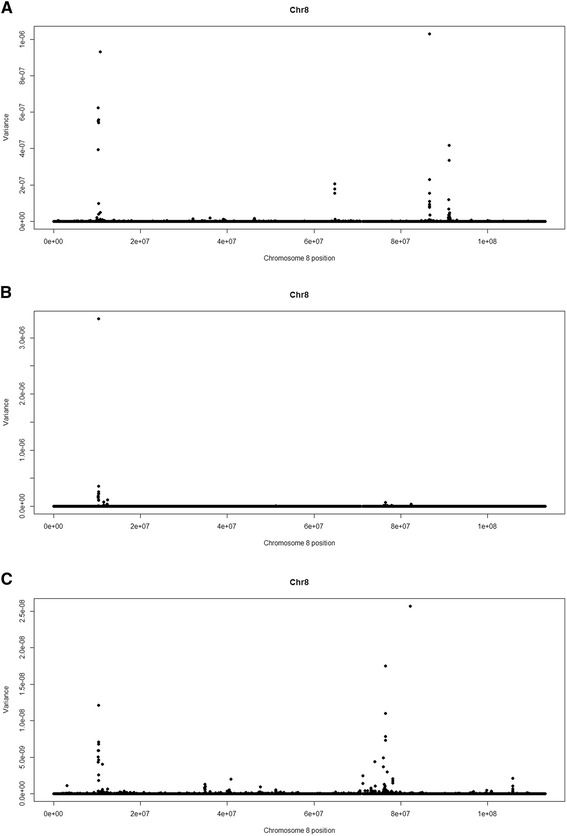
**Plots of additive genetic variance explained by SNPs located on chromosome 8 for A: iron, B: zinc; C: phosphorus.**

Other large-effect QTL were found for muscle contents of Se, Ca and Zn and were respectively located on BTA11 at 22 Mb, BTA8 at 56 Mb and BTA8 at 10 Mb, and explained 3.53, 2.59 and 2.5% of the additive genetic variance in these traits, respectively (Table 2, Figures 2, 3 and 4). These regions contain the following genes: *transmembrane protein 178* (*TMEM178*), *transmembrane channel-like proteins 5 and 7* (*TMC5* and *TMC7*), *ubiquitin specific peptidases* (*USP45* and *USP46*), *sorting nexins 2 and 24* (*SNX2* and *SNX24*) and *scavenger receptors 3 and 5* (*SCARA3* and *SCARA5*). The QTL with the greatest effect identified on BTA8 at 10 Mb, which explained 2.6% of the additive genetic variance of muscle content of Zn, also explained 0.5% of the additive genetic variation for Fe concentration (Figure 5). Allele substitution effects for several SNPs located in this region were in the same direction for muscle contents of Zn and Fe and selection on this QTL could be used to improve the muscle concentrations of both Fe and Zn. In addition, we identified other QTL for Zn content on BTA8 at 11, 12, 76 and 78 Mb. BTA8 harbors QTL that affect muscle contents of Fe, Zn and P (Figure 5). The BTA8 QTL at 72 Mb associated with Fe content is in a region that harbors the s*cavenger receptor class A, member 5* (*SCARA5*) gene, which is involved in cellular iron homeostasis and transport [22], and is a good candidate gene for further studies.

Candidate genes within 100 kb of the most strongly associated SNPs [See Additional file 1: Table S1] were analyzed to search for functional pathways using DAVID. Most of the genes within these regions are involved in signal transduction, signaling pathways via integral proteins, regulation of transcription and metal ion binding [See Additional file 2: Table S2]. Gene clusters associated with QTL for Mg content are involved in transcription regulation and nucleotide binding. Solute carrier family genes were identified as being candidate genes across all mineral tissue contents.

## Discussion

The recovery values and triplicate deviations observed for the mineral analyses were all within acceptable limits [21], which indicate that the measurement methodology for muscle mineral concentration was reliable. The mean concentrations of each mineral, especially for iron (Table 1), were higher than is generally observed for *Bos taurus* cattle [4,23]. This difference may be due to dietary, breed, or age at slaughter differences.

The proportion of phenotypic variance explained by markers, considered to be a measure of heritability, indicates that these traits are at least partially heritable; however, we did not assess the accuracies of genomic predictions and the small animal sample size may have influenced these estimates of heritability. A similar study in the Angus bovine breed reported heritability estimates of 0.48, 0.15 and 0.06 for Fe, Na and Zn concentrations, respectively, when an animal model using the numerator relationship matrix to model identity-by-descent between animals was used to estimate the variance components [9]. In that study, heritability estimates of 0 were obtained for Mg, Mn, P and K muscle content under the same model. However, when the BayesC module (which accounts for genomic relationships among animals) of the GenSel software was used, the proportion of phenotypic variance explained by genome-wide SNPs was 0.37, 0.18, 0.20, 0.12, 0.03, 0.09 and 0.17 for Fe, Mg, Mn, P, K, Na and Zn content, respectively [9]. The differences between the proportions of phenotypic variance explained by marker genotypes and the heritability estimates obtained using an animal model with a pedigree-derived relationship matrix may be due to errors in the pedigree, lack of depth of pedigree to capture additional relationships, or selection of progeny within families for phenotyping (selection on the Mendelian sampling coefficient), all of which are captured in the genomic relationship matrix but not in the numerator relationship matrix.

The detection of similar functional gene clusters for all mineral measurements indicates that similar biological mechanisms appear to affect the contents of different minerals. Some coincident genes and QTL regions [See Additional file 1: Table S1 and Additional file 2: Table S2], located at the centromeric end of BTA8, were found to influence the concentrations of Fe, Zn and P (Figure 5). The *SCARA5* gene (Table 2), which is involved in iron delivery [22] is located in this region of the bovine genome. Other QTL regions that contain genes encoding transmembrane proteins, such as *TMEM178*, *TMC5,* and *TMC7,* were also identified. These genes are intricately involved in the transport of various substrates across cell membranes and are driven by ion gradients [24].

Influx systems of metal ions in cells are strongly regulated by both transcriptional and posttranscriptional control mechanisms [25]. Most candidate genes identified in this study as potentially affecting mineral concentrations are involved in signal transduction, signaling pathways via integral protein membranes, zinc-fingers, regulation of transcription and metal ion binding [See Additional file 2: Table S2], which reinforces the idea that the genetic architecture of muscle mineral concentration probably involves genes that control the transport and homeostasis of ions [1]. Genes that participate in the functions of integral membrane proteins and transmembrane proteins were found in regions that were associated with all mineral concentrations and may transport specific substrates across biological membranes depending on membrane potentials established by ion concentration [26]. Mutations in these genes could cause the abnormal function of these proteins and could influence mineral concentrations.

Iron is needed for several metabolic pathways that operate continuously at the molecular level and are essential to human life [27]. We found a large-effect QTL located on BTA12 that explained 6.5% of the genetic variance for this trait (Table 2); however, the major genomic region identified in the Angus breed and reported by Mateescu et al. [9] was not the same as that detected in this study. The QTL with the largest effect that was detected in Angus cattle was located on BTA15 and explained 4.8% of the genetic variance [9]. In fact, none of the large-effect QTL reported for muscle Fe content identified in Angus cattle [9] co-localized with those found in this study (Table 2). While a QTL at 62 Mb on BTA1 explained 1.5% of the genetic variance for Fe content in Angus cattle, the same region in Nelore cattle explained only 0.05% of the genetic variance. Morris et al. [1] conducted a study in Jersey and Limousin back-cross calves to search for QTL that influenced the amount of minerals in liver, kidney and muscle using microsatellites and the regions of the genome that they identified as harboring large-effect QTL for Fe concentration also did not co-localize with either those detected in this study or those detected by Mateescu et al. [9].

A recent study demonstrated that different genomic regions influence meat quality traits in indicine and taurine cattle [2]. This finding could be due to differences in allele frequencies at the causal mutations and SNPs between these two subspecies and therefore to the extent of LD available to detect QTL [28] or to the divergence between indicine and taurine cattle leading to different QTL architectures. These inconsistencies may be also due to gene-gene (epistasis) or gene-environment interactions [29]. Given the limited number of animals used in this study, it is important to recall that not all the QTL may have been detected, which could also contribute to the differences found between this study and a similar study performed in Angus cattle [9]. Another reason for these inconsistencies could be the difference in resolution of the genotyping platform used. The Illumina BovineHD array used in this study has a considerably higher resolution than the previously used BovineSNP50 assay [9] or microsatellite scans [1], which can allow identification of genomic regions that were not identified in the lower resolution scans.

Genes from the ATP-binding cassette family were found in the region that contained the large-effect QTL for Fe in the Nelore breed on BTA12. ABC proteins transport a number of substrates, including metal ions across the plasma membrane and across intracellular membranes [30]. Numerous genes that are involved in ion transport were candidates for the QTL that affect mineral content. Candidate genes that are involved in sodium transport include *sodium channel as voltage-gated, type III, beta subunit* (*SCN3B*), *renal sodium/dicarboxylate cotransporter* (*SLC13A2*), *sodium channel, voltage-gated, type IV, beta subunit* (*SCN4B*) and in ion channel activity such as *SCN3B*, *chloride intracellular channel 5* (*CLIC5*), *sodium channel, voltage-gated, type IV, beta subunit* (*SCN4B*), and *potassium inwardly-rectifying channel, subfamily J, member 11* (*KCNJ11*) were identified specifically as candidates for Na concentration [See Additional file 1: Table S1].

Many more phenotypic records and further validation in other populations are necessary to accurately estimate the effects of the detected QTL before this information can be efficiently used in animal breeding programs. Despite inconsistencies in the regions identified that harbor large-effect QTL in Angus and Nelore cattle, the functional gene clusters and pathways identified based on candidate gene lists that were generated in both studies indicated that the QTL operate within the same pathways. Some gene networks such as the ATP-binding cassette family genes that play a role in Fe concentration were also reported by Mateescu et al. [9].

Studies on the identification of QTL associated with mineral concentration in different tissues have only recently been performed [1,9] and the mechanisms that control mineral homeostasis remain poorly understood. The increased interest in GWAS is due to the use of molecular markers to improve the accuracy of breeding value estimation and to increase our understanding of the genetic control of important production traits. Moreover, the identification of genes responsible for variation in traits may also provide insight into the biological mechanisms that underlie variation and the likely effects of selection on these polymorphisms [31].

In addition to production efficiency, beef cattle improvement programs should begin to consider traits that influence animal health and that could also benefit to human health [9], such as the mineral content of muscle/beef. High-density SNP genotyping has been used for more than five years to explain variation in quantitative traits of livestock; however, the underlying mechanisms that affect genetic variation are still poorly understood.

## Conclusions

Several genomic regions associated with mineral composition of *longissimus dorsi* muscle were identified across all chromosomes, which reveals the polygenic nature of these traits. We identified a comprehensive list of candidate genes that may underlie the identified QTL regions and that are related to mineral transport and homeostasis [See Additional file 1: Table S1 and Additional file 2: Table S2]. Further validation studies and investigations to identify the causal mutations are necessary. This study provides the first step towards the understanding of the molecular basis of mineral concentration in muscle, which likely involves many genes. The lessons learned from elucidating the genetic architecture of mineral balance in bovine muscle could be used as a model for metabolic diseases in other organisms. This information may also be useful to outline strategies to improve the mineral concentration of muscle and enhance nutritional attributes; however, animal breeding programs should not prioritize these features in the near future, due to the limited current cost-effectiveness.

## Additional files

Additional file 1: Table S1. Summary of enriched gene clusters from DAVID for identified genes for arsenic, calcium, chromium, cobalt, copper, iron, magnesium, manganese, phosphorus, potassium, selenium, sodium, sulfur and zinc in the *longissimus dorsi* muscle of Nelore cattle. Enriched gene clusters from DAVID analysis for candidate genes located in genomic regions associated with each mineral content. Tabs are divided by mineral type. Additional file 2: Table S2. Summary of SNP effects for arsenic, calcium, chromium, cobalt, copper, iron, magnesium, manganese, phosphorus, potassium, selenium, sodium, sulfur and zinc in the *longissimus dorsi* muscle of Nelore cattle. Summary of effects of 224 markers that had the largest posterior probability of being incorporated in the model for each trait. Tabs are divided by mineral type.
